# Addition of Clonidine or Dexmedetomidine With Bupivacaine to Prolong Caudal Analgesia in Children Undergoing Infraumbilical Surgery

**DOI:** 10.7759/cureus.23110

**Published:** 2022-03-13

**Authors:** Saurov Gogoi, Diganta Saikia, Sandeep Dey

**Affiliations:** 1 Anesthesiology, Assam Medical College and Hospital, Dibrugarh, IND; 2 Anesthesiology, Tezpur Medical College and Hospital, Tezpur, IND; 3 Anesthesiology, Jorhat Medical College and Hospital, Jorhat, IND

**Keywords:** postoperative pain, infraumbilical surgery, caudal analgesia, clonidine, dexmedetomidine, children

## Abstract

Introduction

Clonidine, a selective α2 adrenergic receptor agonist, combined with caudally administered bupivacaine, is frequently used in children to prolong the duration of postoperative analgesia following infraumbilical surgery. On the other hand, dexmedetomidine is highly selective and has a greater affinity toward α2 adrenergic receptors, especially toward its α2a subtype, accountable for more analgesic and hypnotic effects than clonidine.

Aims and objectives

We designed a prospective, double-blinded, randomized controlled trial to compare the analgesic efficacy and adverse effects of clonidine and dexmedetomidine when combined with bupivacaine for caudal analgesia in children undergoing infraumbilical surgeries.

Materials and methods

A total of 60 children aged one to eight years were randomly assigned into two different groups in a double-blinded manner. Following induction of general anesthesia, every patient received a single dose of caudal bupivacaine 0.25% (0.75 ml/kg) mixed with either clonidine (1 µ/kg) in normal saline or dexmedetomidine (1 µ/kg) in normal saline. We noted the hemodynamic variables and postoperative sedation scores. Duration and quality of postoperative analgesia and the number of rescue analgesic drug doses required were recorded during the first 24 hours postoperatively. We also observed the patients for any adverse effects to the study drugs.

Result

Adding dexmedetomidine to caudally administered bupivacaine significantly increased the duration of analgesia (15 ± 0.78 hours) and decreased the need for rescue drug doses than the addition of clonidine to bupivacaine (9.63 ± 1.95 hours) in children undergoing infraumbilical surgeries. Incidences of hemodynamic changes or other side effects were comparable between patients of two groups.

Conclusion

The addition of dexmedetomidine to caudally administered bupivacaine in children undergoing infraumbilical surgeries may provide a longer duration of analgesia than the addition of clonidine, with less requirement of rescue analgesic doses and without any significant differences in the hemodynamic parameters or other side effects.

## Introduction

Pain experienced by children and infants often goes unrecognized, even neglected or undertreated, because of the operational definition of pain requiring self-reporting [1,2]. Also, compared to adults, children receive fewer, less frequent, and smaller doses of potent opioids [3]. Caudal epidural analgesia in children undergoing abdominal and lower limb surgery is a novel procedure to combat intraoperative and postoperative pain [4]. The most commonly used drug is long-acting local anesthetic bupivacaine. However, single-shot caudal bupivacaine produces analgesia for a short duration, lasting only four to eight hours [5]. Thus, various adjuvant drugs, including α2 adrenergic receptor agonists, are used to prolong the duration of action of epidural bupivacaine [6]. Clonidine, a selective α2 adrenergic receptor agonist, when added to caudally administered bupivacaine significantly prolongs the median duration of analgesia and reduces the analgesic requirement during the first 24 hours postoperatively in children following infraumbilical surgery compared to bupivacaine alone [7]. Dexmedetomidine, a highly selective α2 adrenergic receptor agonist, has an 8-10 fold greater affinity for α2 adrenergic receptors without significant effects on α1 adrenergic receptors [8]. As such, the higher selectivity of dexmedetomidine towards α2a subtype of α2 adrenergic receptors accounts for more significant analgesic and hypnotic effects than clonidine [9].

We designed a prospective trial to compare the analgesic efficacy of clonidine and dexmedetomidine when added to caudally administered bupivacaine in children undergoing infraumbilical surgery (primary objective). We compared the analgesic efficacy by recording the duration between caudal injection and administration of the first dose of postoperative rescue analgesia. Also, we noted the number of doses of rescue analgesia that were required. Our secondary objective was to compare the degree of variation of hemodynamic parameters and incidences of any other side effects, if any, between two groups of children.

## Materials and methods

After obtaining approval from the Institutional Ethics Committee (Human) (registration number: ERC/636/Inst/AS/2014; approval number: AMC/EC/PG 2497; dated April 18, 2018), we conducted a prospective, double-blinded, randomized controlled trial in Assam Medical College and Hospital, Assam (India). We obtained written informed consent from every child's parent/legal guardian. We included 60 children, aged one to eight years, with the American Society of Anesthesiologists' (ASA) physical status I or II, undergoing various elective infraumbilical surgeries from July 1, 2018, to June 30, 2019.

We excluded children whose parents/guardians refused to give consent; children with a history of allergy to study drugs; those with bleeding or coagulation disorder or developmental delay; and those with evidence of infection over the lower back or anatomical malformation at the puncture site. We also excluded patients who required sedative premedication or who underwent emergency surgery.

**Figure 1:**
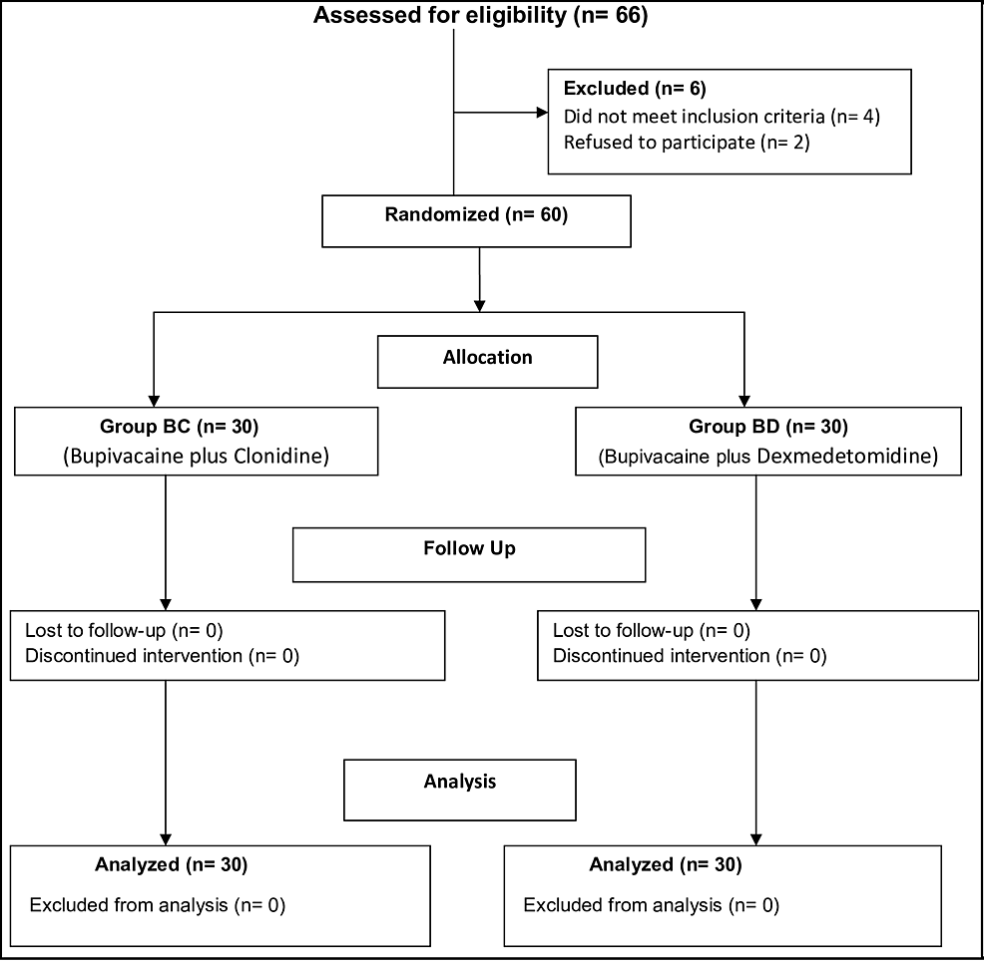
Consort flow diagram.

We randomly assigned the patients into groups using computer-generated random numbers. Each group consisted of 30 patients and was named group BC (group of patients receiving bupivacaine with clonidine) and group BD (group of patients receiving bupivacaine with dexmedetomidine) (Figure 1). In the preoperative area, a team member confirmed the child's name, age, sex, weight, and type and site of the proposed surgical procedure. All patients were induced with 50% nitrous oxide and 50% oxygen with 8% sevoflurane in a single-step manner. On attaining an adequate depth of anesthesia, we secured an intravenous line and injected glycopyrrolate 4 mcg/kg followed by fentanyl 1 mcg/kg intravenously. After that, we placed the children in the left semi-prone position and a senior anesthesiologist performed a single-shot caudal epidural block using a 23 G hypodermic needle under aseptic conditions. Blinding was ensured by engaging a different anesthesiologist not involved in the study to prepare the drug (in a standardized 5-ml syringe). Needle placement in the caudal space was confirmed using the loss of resistance technique.

Group BC patients received bupivacaine 0.25%, 0.5-0.75 ml/kg (as par Armitage formula) mixed with 1 µ/kg of clonidine in 1 ml normal saline and group BD patients received bupivacaine 0.25%, 0.5-0.75 ml/kg mixed with 1 µg/kg of dexmedetomidine in 1 ml normal saline [10]. We calculated the dose of bupivacaine as 0.5-0.75 ml/kg body weight based on the type of surgical procedure performed on the particular child. We then placed the patients supine and inserted a laryngeal mask airway (LMA Supreme) of appropriate size for the child's weight to maintain the airway. We maintained the depth of anesthesia using sevoflurane (1-3%) in nitrous oxide and oxygen (50:50), with assisted ventilation via the Jackson Rees circuit. The skin incision was allowed following 10-15 minutes of placing the caudal block. The caudal block was accepted adequate if heart rate (HR) and mean arterial blood pressure (MAP) did not increase more than 20% of baseline value after skin incision. After the surgery, we stopped sevoflurane and nitrous oxide, removed the LMA, and continued oxygenation via face mask oxygenation for three to five minutes. The patients were shifted to the post-anesthetic care unit (PACU) after he/she became aroused.

We monitored the patients continuously during the intraoperative period using ASA perioperative monitoring guidelines. HR and non-invasive blood pressure (NIBP) were recorded before and after anesthesia induction, before caudal block placement, after skin incision, and after that, every 15 minutes until the surgery was over. Incidences of intraoperative hypotension (fall in NIBP > 20% from baseline) and bradycardia (fall in HR > 20% from baseline) were recorded. We replaced the fluid deficit and perioperative fluid and blood loss using lactated Ringer's solution and blood as applicable. We documented the anesthetic time (time from induction to the completion of surgery), emergence time (time from completion of surgery to eye opening on command), and delayed emergence (taking >20 minutes from completion of surgery to transfer to PACU).

We continuously monitored the patients' hemodynamic parameters and oxygen saturation (SpO2) postoperatively. We assessed the pain intensity using pediatric observational FLACC (face, legs, activity, cry, consolability) score upon patient admission in PACU [11]. After that, the assessment was done every hour until six hours, followed by every three hours, till 12 hours, and then every six hours till 24 hours until the first dose of rescue analgesia was necessary. The patients received the first rescue analgesia (oral paracetamol 15 mg/kg) when the FLACC pain score was four or more [12] (range being 0 to 10). We recorded the duration between caudal injection and administration of the first dose of postoperative rescue analgesia (primary outcome of our study). Also, we noted the number of doses of rescue analgesia that were required.

Secondary outcomes of our study were the duration of the motor block, postoperative sedation, and other side effects of the study drug combinations. We assessed the motor block on the child's awakening by using a modified Bromage scale [13]. However, younger children who could not move their legs on command were stimulated by tapping on the legs and feet. We assessed the level of sedation by Ramsay sedation scale (RSS) [14] at 15, 30, and 60 minutes after removal of LMA and after that hourly until the RSS score became 1 in all children. Duration of postoperative sedation was considered from the LMA removal until RSS was 2 or less. We observed instances of postoperative respiratory depression (SpO2 < 95% or respiratory rate < 10/m), hypotension, bradycardia, and postoperative nausea and vomiting (PONV) requiring treatment. We treated episodes of hypotension and bradycardia with fluid bolus and injection atropine, respectively, and PONV with intravenous ondansetron. We also noted the duration of PACU stay, time to the first micturition after caudal injection, time of initiation of feeding, and time to discharge.

Sample size calculation

We calculated the sample size using Power Analysis and Sample Size (PASS) 15.0 software (NCSS, Kaysville, UT). The primary endpoint was the time required to a FLACC score ≥ 4 after administering the study drug. We determined the number of subjects required in each group using power calculation with data obtained from a pilot study. The expected mean duration of analgesia for the dexmedetomidine and clonidine groups was 770.21 (91.19) minutes and 580.02 (40.38) minutes, respectively. Hence, a sample size of 24 would detect a 240 minutes difference in the duration of analgesia between the groups (with α = 0.05, β = 0.20, and power of 80%). Therefore, we included 30 subjects in each group to replace any dropouts.

Randomization (sequence generation/allocation concealment mechanism/implementation blinding)

Using computer-generated random numbers, an independent senior resident made random allocation cards for 60 selected patients and divided the patients into two equal groups of 30 each. The groups were named group BC (group of patients receiving bupivacaine with clonidine) and group BD (group of patients receiving bupivacaine with dexmedetomidine). Another independent senior resident used SNOSE (sequentially numbered, opaque, sealed, and stapled en­velopes) method to conceal the allocation sequence from the researcher, enrolling and assessing participants and thereby the participants and the investigators. The envelopes were opened sequentially just before the in­jection by an independent nurse, who prepared the injection as mentioned in the card inside for that particular patient and handed over the syringe to the anesthetist performing the study procedure. The anesthetist recorded the input date, time, patient ID, and the results after the procedure on the envelope or another sheet inside the envelope. The envelope was sealed and preserved in a secured place for analysis by the principal investigating officer and future references.

Statistical analysis

Data collected were entered into a computer-based spreadsheet for analysis using SPSS statistical software (version 20.0; IBM Corporation, Armonk, NY). We presented the numerical variables (e.g., age, weight, HR, and blood pressure) as mean and standard deviation and categorical variables (e.g., sex and adverse events) as frequency (%). We used the Student's t-test for numerical values and the chi-square test for categorical values. The p-value < 0.05 was considered statistically significant.

## Results

**Figure 2:**
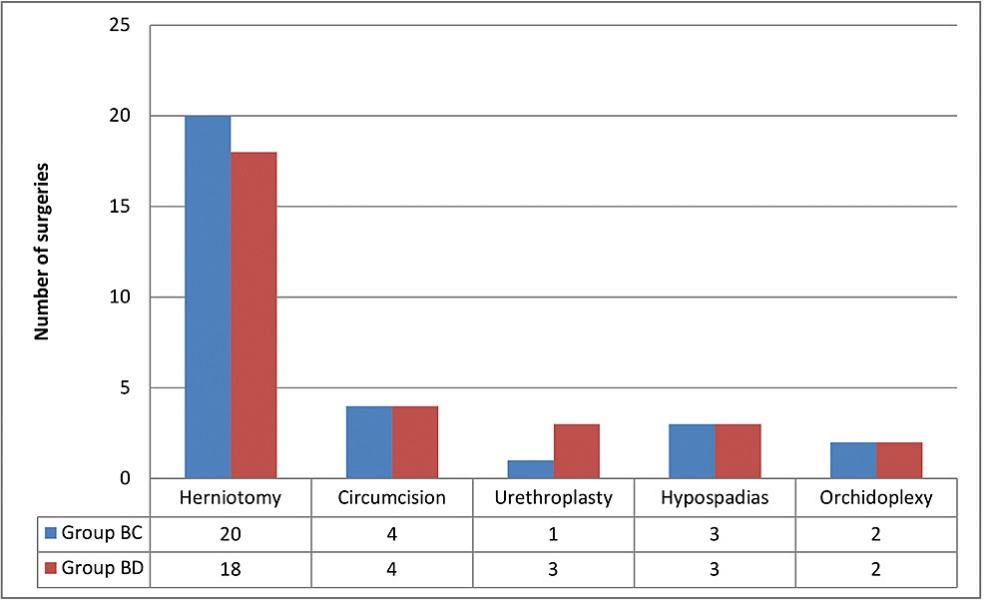
Types of surgeries. BC, bupivacaine with clonidine; BD, bupivacaine with dexmedetomidine.

We assessed 66 patients for eligibility. After excluding six patients (four patients who did not meet inclusion criteria and two patients who did not consent), we included 60 patients in the study (Figure 1). Table 1 demonstrates the demographic parameters of the participants. We found no statistically significant difference amongst the study participants in their demographic parameters. Figure 2 enumerates the various infraumbilical surgeries performed amongst the study participants in the two groups, and Table 2 shows the duration (in minutes) that those procedures lasted. Our study mainly consisted of cases involving herniotomy, circumcision, urethroplasty, orchidopexy, and hypospadias repair. Of those 60 patients in the two groups, 46 patients had a surgery duration of 0-30 minutes, amongst four patients, it was 31-60 minutes, and in the rest 10 patients, the surgery lasted 61-90 minutes.

**Table 1:** Demographic parameters of the study participants. SD, standard deviation; BC, bupivacaine with clonidine; BD, bupivacaine with dexmedetomidine.

Variables	Group		P-value
	BC (n = 30)	BD (n = 30)	
Age (years), mean ± SD	5.13 ± 1.40)	5.20 ± 2.04	0.833
Weight (kg), mean ± SD	17.03 ± 7.04	19.46 ± 7.68	0.206
Sex, n (%)			
Male	28 (93.4%)	26 (86.7%)	0.389
Female	2 (6.6%)	4 (13.3%)	

**Table 2:** Duration of surgery. BC, bupivacaine with clonidine; BD, bupivacaine with dexmedetomidine.

Duration of surgery (minutes)	Group BC		Group BD		Total	
	Number	Percentage	Number	Percentage	Number	Percentage
0-30	26	56.5%	20	43.5%	46	100%
31-60	0	0.0%	4	100.0%	4	100%
61-90	4	40.0%	6	60.0%	10	100%
Total	30	50.0%	30	50.0%	60	100%

Tables 3, 4 give a comparative analysis of the hemodynamic parameters amongst the participants in the two groups. The mean baseline HR of the patients in groups BC and BD was 109.87 (± 9.03) beats/min and 105.97 (±10.69) beats/min, respectively, and the difference was not statistically significant. The maximum decrease in the patients' HR in groups BC and BD was 106.83 (±8.96) beats/min and 102.80 (±7.07) beats/min, respectively. It was also not statistically significant (Table 3).

**Table 3:** Hemodynamic data (heart rate). SD, standard deviation; BC, bupivacaine with clonidine; BD, bupivacaine with dexmedetomidine.

Heart rate (beats/min)	Group		P-value
	BC	BD	
	Mean ± SD	Mean ± SD	
Baseline	109.8 ± 9.03	105.97 ± 10.69	0.132
Maximum decrease recorded	106.8 ± 8.96	102.80 ± 7.07	0.116

**Table 4:** Hemodynamic data (MAP). SD, standard deviation; BC, bupivacaine with clonidine; BD, bupivacaine with dexmedetomidine; MAP, mean arterial blood pressure.

MAP (mmHg)	Group		P-value
	BC	BD	
	Mean ± SD	Mean ± SD	
Baseline	74.47 ± 1.717	74.93 ± 2.083	0.348
Maximum decrease recorded	73.70 ± 3.90	74.60 ± 4.10	0.388

The baseline MAP of the patients in groups BC and BD was 74.47 (±1.717) mmHg and 74.93 (±2.083) mmHg, respectively, and the difference between the groups was not significant. Similarly, the maximum decrease in MAP of the patients in groups BC and BD was 73.70 (±3.90) mmHg and 74.60 (±4.10) mmHg, respectively, and the difference was also not statistically significant (Table 4).

Table 5 shows the duration of analgesia with the different study drugs. The mean duration of analgesia in group BC was 9.63 (±1.95) hours, while it was 15 (±0.78) hours in group BD. The difference in the mean duration of analgesia between the two groups was statistically significant.

**Table 5:** Duration of analgesia. SD, standard deviation; BC, bupivacaine with clonidine; BD, bupivacaine with dexmedetomidine.

Duration of analgesia (hours)	Group		P-value
	BC	BD	
Mean ± SD	9.63 ± 1.95	15 ± 0.78	0.000
Minimum-maximum	0-11	14-16	

Table 6 gives a comparative analysis of the mean FLACC score amongst the participants in the two groups. The mean FLACC pain score was significantly lower in group BD compared to group BC at the end of one hour, two hours, six hours, and 24 hours (0.07 ± 0.254 versus 0.87 ± 0.346, 0.57 ± 0.504 versus 1.80 ± 0.610, 2.0 ± 0.0 versus 2.87 ± 0.346, and 0 ± 0 versus 3.67 ± 1.322, respectively, p = 0.00).

**Table 6:** Comparison of FLACC pain scores. SD, standard deviation; BC, bupivacaine with clonidine; BD, bupivacaine with dexmedetomidine; FLACC, face, legs, activity, cry, consolability.

FLACC score (at end of)	Group BC	Group BD	P-value
	Mean ± SD	Mean ± SD	
0 minutes	0 ± 0	0 ± 0	-
1 hour	0.87 ± 0.346	0.07 ± 0.254	0.00
2 hour	1.80 ± 0.61	0.57 ± 0.504	0.00
6 hour	2.87 ± 0.346	2.0 ± 0.0	0.00
12 hour	3.07 ± 0.45	3 ± 0.0	0.42
24 hour	3.67 ± 1.322	0 ± 0	0.00

Table 7 shows the sedation score (RSS score) of the participants in the two groups. The mean RSS score was significantly lower in group BC compared to group BD at the end of two hours and three hours (2 ± 0 versus 4.03 ± 0.183 and 2 ± 0 versus 2.43 ± 0.568, respectively, p = 0.00).

**Table 7:** Comparison of RSS scores. SD, standard deviation; BC, bupivacaine with clonidine; BD, bupivacaine with dexmedetomidine; RSS, Ramsay sedation scale.

RSS score (at end of)	Group BC	Group BD	P-value
	Mean ± SD	Mean ± SD	
0 minutes	5 ± 0	6 ± 0	-
1 hour	4 ± 0	5 ± 0	-
2 hour	2 ± 0	4.03 ± 0.18	0.00
3 hour	2 ± 0	2.43 ± 0.56	0.00
6 hour	2 ± 0	2 ± 0	-
12 hour	2 ± 0	2 ± 0	-

Table 8 gives an overview of the number of rescue analgesia required amongst the study participants in the two groups. In group BC, 20 (66.7%) participants needed one dose of rescue analgesia, six (20.0%) participants required two doses, and one (3.3%) participant required three doses of rescue analgesia. In group BD, none of the patients required any amount of rescue analgesia. This difference in the need for rescue analgesia amongst groups BC and BD participants was statistically significant (p < 0.00).

**Table 8:** Number of rescue analgesia doses required. BC, bupivacaine with clonidine; BD, bupivacaine with dexmedetomidine.

Number of doses	Group BC		Group BD		P-value
	Number	Percentage	Number	Percentage	
0	3	10.0	30	100.0	<0.000 (chi-square test)
1	20	66.7	0	0	
2	6	20.0	0	0	
3	1	3.3	0	0	
Total	30	-	30	-	

Table 9 depicts the side effects noted with the study drugs. Amongst the participants in group BC, three (10.0%) complained of nausea, and two (6.6%) complained of vomiting. On the other hand, amongst the participants in group BD, two (6.6%) complained of nausea, two (6.6%) complained of vomiting, and one (3.3%) complained of urinary retention. We found no statistically significant difference in the incidence of side effects amongst the participants in the two groups.

**Table 9:** Incidence of postoperative side effects. BC, bupivacaine with clonidine; BD, bupivacaine with dexmedetomidine.

Postoperative side effect	Group BC (n = 30)		Group BD (n = 30)		P-value
	Number	Percentage	Number	Percentage	
Nausea	3	10.0	2	6.6	0.64
Vomiting	2	6.6	2	6.6	-
Urinary retention	0	-	1	3.3	0.31
Pruritus	0	-	0	-	-
Convulsion	0	-	0	-	-

## Discussion

Postoperative pain management has become an integral part of pediatric anesthesia. The caudal epidural is the most common regional anesthesia technique employed for providing anesthesia and analgesia in children undergoing infraumbilical surgeries. In our study, caudal epidural block using 0.25% bupivacaine in combination with clonidine and dexmedetomidine was conducted in 60 patients aged one to eight years, with ASA grades I and II, undergoing various infraumbilical surgeries.

We found no significant difference in age, sex, and weight between the two groups. Our study had more male participants in both groups (group BC = 93.4% versus group BD = 86.7%). Most of the patients had undergone surgical procedures like herniotomy, circumcision, orchidopexy, and urethroplasty and were comparable between the groups. Duration of surgery was also similar in both the groups and was statistically not significant.

We noted that group BC's mean baseline HR was 109.87 (±9.03) beats/min and 105.97 (±10.69) beats/min in group BD. The maximum decrease in HR in group BC was 106.83 (±8.96) beats/min, while in group BD, it was 102.80 (±7.07) beats/min. Our results were consistent with works by El-Hennawy et al. [15], Ganeshnavar et al. [16], and Raval and Kartik [17]. They also reported no significant changes in HR amongst the study participants.

The mean baseline MAP of the participants in groups BC and BD was 74.47 (±1.717) and 74.93 (±2.083) mmHg, respectively. The maximum decrease in MAP of the participants in groups BC and BD was 73.70 (±3.90) and 74.60 (±4.10) mmHg, respectively. Both these parameters were not statistically significant. This decrease in MAP was similar to the findings by El-Hennawy et al. [15], Parameswari et al. [18], Goswami et al. [19], Ganeshnavar et al. [16], and Raval and Kartik [17], who also reported insignificant changes in MAP among their study groups.

We found that the duration of caudal analgesia was significantly higher in the group receiving bupivacaine-dexmedetomidine (15 ± 0.78 hours) than the group receiving bupivacaine-clonidine (9.63 ± 1.95 hours). EL-Henawy et al. [15], Ganeshnavar et al. [16], and Raval and Kartik [17] also found that the group receiving bupivacaine-dexmedetomidine had a longer duration of analgesia compared to the group receiving bupivacaine-clonidine. In other studies comparing bupivacaine with bupivacaine and dexmedetomidine by Saadawy et al. [20], Goyal et al. [21], and Goswami et al. [19], children in the bupivacaine and dexmedetomidine group had a duration of analgesia significantly longer than bupivacaine alone. Also, in studies comparing bupivacaine with bupivacaine and clonidine, Parameswari et al. [18], Hooda [22], Meghani et al. [23], Bhatia et al. [24], and Rajan et al. [25] found that children receiving bupivacaine and clonidine had a significantly longer duration of analgesia than bupivacaine alone.

Our study found that the mean FLACC pain score was significantly lower in the group receiving bupivacaine-dexmedetomidine than bupivacaine-clonidine at the end of one hour, two hours, six hours, and 24 hours. Similar to our study, El-Hennawy et al. [15] found that patients in the bupivacaine-dexmedetomidine group had significantly lower FLACC scores at four hours postoperatively than patients in the bupivacaine-clonidine group. Parameswari et al. [18] found significantly lower FLACC scores from two to six hours postoperatively amongst patients in the bupivacaine-clonidine group than patients in the bupivacaine group. Goyal et al. [21] found that the mean FLACC pain score was significantly less in patients belonging to the bupivacaine-dexmedetomidine group for 12 hours postoperatively than patients in the bupivacaine group. Hooda et al. [22] found significantly lower FLACC scores at one hour and four hours postoperatively amongst patients belonging to the bupivacaine clonidine group than patients in the bupivacaine group.

We found that the mean RSS score was significantly lower at the end of two hours and three hours in the group receiving bupivacaine-clonidine compared to the group receiving bupivacaine-dexmedetomidine. Similar to our study, Saadawy et al. [20] and Goswami et al. [19] found that the mean duration of sedation was significantly prolonged amongst patients belonging to the bupivacaine-dexmedetomidine group compared to patients in the bupivacaine group. Also, Hooda et al. [22], Bhatia et al. [24], and Meghani et al. [23] found that the median sedation score was higher in patients receiving caudal bupivacaine-clonidine compared to those receiving only bupivacaine.

Amongst children who received caudal bupivacaine-clonidine, 20 patients (66.7%) required a single dose, six patients (20.0%) required two doses, and one (3.3%) patient required three doses of supplemental (rescue) analgesic. No patient required rescue analgesia in the group receiving caudal bupivacaine-dexmedetomidine. Ganeshnavar et al. [16] also found that the time for rescue analgesia was statistically prolonged in the bupivacaine-dexmedetomidine group (17.6 ± 2.9 hours) when compared to the bupivacaine-clonidine group (10.1 ± 3.2 hours) (p < 0.05). In other studies by Saadawy et al. [20] and Goyal et al. [21], they found that children receiving caudal bupivacaine-dexmedetomidine required significantly less dose/s of additional rescue analgesia during the first 12-24 hours postoperatively, compared to children receiving bupivacaine alone. Parameswari et al. [18], Meghani et al. [23], Bhatia et al. [24], and Rajan et al. [25] found that the requirement of rescue analgesia for 12-24 hours postoperatively was significantly less in the group receiving bupivacaine-clonidine compared to the group receiving plain bupivacaine.

Among the participants, who received caudal bupivacaine-clonidine, three patients (10.0%) complained of nausea, and two patients (6.6%) complained of vomiting. Amongst the participants who received bupivacaine-dexmedetomidine, two patients (6.6%) complained of nausea, two patients (6.6%) complained of vomiting, and one patient (3.3%) complained of urinary retention. There was no statistical difference in the incidence of side effects amongst the two groups. Saadawy et al. [20] found that the incidence of vomiting, time for first micturition, and spontaneous leg movements were not significantly different among the groups receiving caudal bupivacaine-dexmedetomidine or bupivacaine-clonidine. Goswami et al. [19] and Gupta et al. [26] also found that the incidence of vomiting and urinary retention was equal between the groups receiving bupivacaine-dexmedetomidine or plain bupivacaine. On the contrary, Goyal et al. [21] found that the incidence of nausea and vomiting was higher in the group receiving plain bupivacaine compared to the group receiving bupivacaine-dexmedetomidine. Meghani et al. [23] and Bhatia et al. [24] also found that nausea and vomiting were higher in children who received bupivacaine-clonidine than plain bupivacaine.

Limitations of the study

Our study had a few significant limitations. Our sample size was small. Due to the absence of bedside ultrasonography, we performed a single-shot caudal epidural block using the loss of resistance technique. We excluded infants and older children (aged more than eight years). We also excluded emergency surgery cases and patients with ASA class III or higher.

## Conclusions

Pain in children often goes undiagnosed and undertreated. So, a method of delivering adequate postoperative analgesia is of utmost importance in children. We designed a prospective, double-blinded, randomized controlled trial comparing the efficacy of two different drugs (clonidine and dexmedetomidine) added to caudal bupivacaine. We conclude that adding dexmedetomidine to caudally administered bupivacaine in children undergoing infraumbilical surgeries provides analgesia longer than clonidine, with less requirement of rescue analgesia and without any significant differences in the hemodynamic parameters or side effects.
